# Genomic Selection in Tropical Forage Grasses: Current Status and Future Applications

**DOI:** 10.3389/fpls.2021.665195

**Published:** 2021-04-30

**Authors:** Rosangela M. Simeão, Marcos D. V. Resende, Rodrigo S. Alves, Marco Pessoa-Filho, Ana Luisa S. Azevedo, Chris S. Jones, Jorge F. Pereira, Juarez C. Machado

**Affiliations:** ^1^Embrapa Gado de Corte, Campo Grande, Brazil; ^2^Embrapa Café, Universidade Federal de Viçosa, Viçosa, Brazil; ^3^Instituto Nacional de Ciência e Tecnologia do Café, Universidade Federal de Viçosa, Viçosa, Brazil; ^4^Embrapa Cerrados, Brasília, Brazil; ^5^Embrapa Gado de Leite, Juiz de Fora, Brazil; ^6^International Livestock Research Institute, Nairobi, Kenya

**Keywords:** apomixis, brachiaria, elephant grass, forage breeding, Guinea grass, marker-assisted selection, polyploidy

## Abstract

The world population is expected to be larger and wealthier over the next few decades and will require more animal products, such as milk and beef. Tropical regions have great potential to meet this growing global demand, where pasturelands play a major role in supporting increased animal production. Better forage is required in consonance with improved sustainability as the planted area should not increase and larger areas cultivated with one or a few forage species should be avoided. Although, conventional tropical forage breeding has successfully released well-adapted and high-yielding cultivars over the last few decades, genetic gains from these programs have been low in view of the growing food demand worldwide. To guarantee their future impact on livestock production, breeding programs should leverage genotyping, phenotyping, and envirotyping strategies to increase genetic gains. Genomic selection (GS) and genome-wide association studies play a primary role in this process, with the advantage of increasing genetic gain due to greater selection accuracy, reduced cycle time, and increased number of individuals that can be evaluated. This strategy provides solutions to bottlenecks faced by conventional breeding methods, including long breeding cycles and difficulties to evaluate complex traits. Initial results from implementing GS in tropical forage grasses (TFGs) are promising with notable improvements over phenotypic selection alone. However, the practical impact of GS in TFG breeding programs remains unclear. The development of appropriately sized training populations is essential for the evaluation and validation of selection markers based on estimated breeding values. Large panels of single-nucleotide polymorphism markers in different tropical forage species are required for multiple application targets at a reduced cost. In this context, this review highlights the current challenges, achievements, availability, and development of genomic resources and statistical methods for the implementation of GS in TFGs. Additionally, the prediction accuracies from recent experiments and the potential to harness diversity from genebanks are discussed. Although, GS in TFGs is still incipient, the advances in genomic tools and statistical models will speed up its implementation in the foreseeable future. All TFG breeding programs should be prepared for these changes.

## Introduction

The tropics are home to about a third of the world’s population, accounting for 36% of the Earth’s landmass, and where most of the global demographic increase takes place (Morales, 2009). The tropical region is the center of origin and domestication of many of the world’s most important food crops and is responsible for 50% beef production and 40% milk production worldwide (Morales, 2009; Alexandratos and Bruinsma, 2012). Despite the huge importance of the tropical region, there is an evident gap in technological development between the tropical nations and industrialized temperate countries. The tropical region has great potential to meet the growing global demand for food requirements with intensification through improved management and technologies. One important step toward intensification is the acceleration of breeding programs.

Plant breeding has evolved from a rudimentary process in its early stages to a modern and sophisticated system in the past few decades. The rediscovery of Mendel’s laws of genetics in 1900 is one of the pillars of modern plant breeding (Hallauer, 2011), which can also count on modern techniques such as high-throughput sequencing, bioinformatics, and automated phenotyping (Barabaschi et al., 2016). Breeding pipelines can apply these techniques to increase the rates of genetic gain taking into account the parameters found in the “breeder’s equation” (Lush, 1937; Eberhart, 1970), which states that the genetic gain is directly proportional to the accuracy of the observed phenotype in relation to the true phenotype and genotype, selection intensity, and genetic variation, but inversely proportional to the time of the breeding cycle. Manipulating variables in the “breeder’s equation” can increase genetic gain as well as reduce the timeframe to develop new cultivars (Pereira et al., 2018).

Pastures are the main food source for animal feeding in the tropics, as in Brazil, where approximately 90% of the livestock are solely grass-fed (Silva et al., 2016). An increase in productivity and quality of tropical forage grasses (TFGs) will have a significant impact on livestock production. Although, cattle and buffaloes already contribute to the largest proportion of global animal protein supply, increased quantities of milk and beef are necessary due to growing demands. Production will have to increase by 57% for beef and 48% for milk by 2050 compared to that in 2005, as projected by the FAO (Alexandratos and Bruinsma, 2012), while other estimates indicate that the global demand for livestock products will double by 2050 (Bajželj et al., 2014; Rao et al., 2015). This higher production needs to take into account scenarios where the land destined for pastures may have to be reduced, as has been happening in Brazil (IBGE, 2016). Efforts to breed TFGs focus on increasing productivity and quality while also reducing losses due to biotic and abiotic stresses. However, breeding efforts have been hindered by many features of tropical forages that render the implementation of more dynamic breeding programs difficult. TFGs encompass perennial monocotyledonous plants from the family Poaceae, mostly polyploid, with a C4 photosynthetic pathway and showing both sexual and apomictic reproductive systems. Breeding programs of TFGs face challenges such as different ploidy levels and reproductive modes, evaluation of perennial plants over different cuts, distribution of efforts among different species, the evaluation of traits being laborious and expensive, and most breeding programs being held by public institutions. The development and release of a new cultivar can take up to 10 years (Jank et al., 2014).

Genomic selection (GS) offers the opportunity to increase agricultural production and reduce the breeding interval cycle to at least half of the conventional time (Crossa et al., 2017). Reduction of the breeding cycle is the main advantage of GS in forage breeding (Simeão-Resende et al., 2014). GS and genome-wide association studies (GWAS) have enormous potential for use in the selection of complex traits such as yield, disease, and insect resistance, facilitating the rapid selection of new cultivars to meet the future demand for food and fodder (Heffner et al., 2010; Talukder and Saha, 2017). However, breeding programs of TFGs are still behind those of grain and fiber crops, and even those of temperate/sub-tropical forages, regarding the application of genomic tools as a strategy to accelerate cultivar development. Challenges in applying GS in tropical forages include designing and obtaining adequately sized training populations; developing high-quality, low-cost, and reproducible marker panels; dealing with polyploidy; and gaining knowledge of the genetic architecture of target traits.

This article provides an overview of GS in TFGs focusing on the current scenario, recent advances, and prospects for the effective application of tools and strategies to accelerate TFG breeding. We have focused on elephant grass (*Cenchrus purpureus* syn. *Pennisetum purpureum*), Guinea grass (*Megathyrsus maximus* syn. *Panicum maximum*), and brachiaria (*Urochloa brizantha* syn. *Brachiaria brizantha*, *U. decumbens* syn. *B. decumbens*, and *U. ruziziensis* syn. *B. ruziziensis*), which account for most of the pastures in many parts of the world, including Africa, Asia, Australia, and Latin America. Specific features of breeding programs, availability of genomic resources, statistical methods for GS, and gaps in the application of GS in TFG breeding are discussed here. This discussion is essential for the initiation and practical implementation of GS in TFG breeding programs.

## TFG Breeding

TFG breeding began relatively recently (Valle et al., 2009). For example, EMBRAPA, the Brazilian Agricultural Research Corporation, started its breeding programs for *Urochloa* and *Megathyrsus* in the 1980s (Jank et al., 2014), while the elephant grass breeding program only began in 1991 (Pereira et al., 2017). One of the first steps toward an effective breeding program is the compilation of a germplasm collection. Approximately 17,000 accessions of TFGs have been preserved in the primary global germplasm banks, such as CIAT, EMBRAPA, IBERS, ICARDA, ILRI, SARDI, and USDA, where *Urochloa* spp., *Cenchrus* spp., and *Megathyrsus* spp. correspond to most of the accessions alongside *Digitaria* spp. and *Paspalum* spp. The genetic variability maintained in these germplasm banks is invaluable, and these banks offer genetic resources adapted to varied edaphoclimatic conditions and diverse purposes. However, there is no corresponding use of this variability during crossings in practical breeding programs. This indicates that these collections are not being used to their full potential, although, initiatives of germplasm exchange between institutions have been taken to increase genetic variability that can be used in breeding programs (Negawo et al., 2018; Habte et al., 2020). Better characterization of germplasm collections will enable quicker utilization to meet the dynamic demands of the production sector with the emergence of diverse limitations due to climate, pests, or changes in production systems.

**FIGURE 1:**
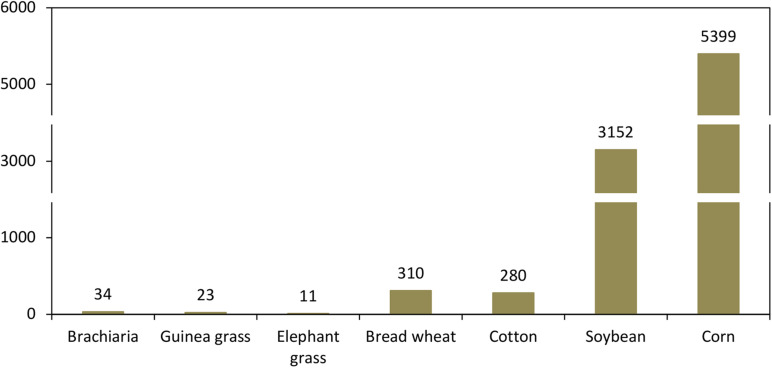
Number of cultivars of brachiaria, Guinea grass, and elephant grass registered in Brazil in comparison with grain and fiber crops. The list was obtained from the National Cultivar Registry Data Bank (*Registro Nacional de Cultivares – RNC*) that is a requirement from the Brazilian Ministry of Agriculture, Livestock and Food Supply since 1997. The numbers shown here were retrieved on 19 November 2020 (http://sistemas.agricultura.gov.br/snpc/cultivarweb/cultivares_registradas. php). Because the difference in the number of cultivars is high, the Y-axis has been adjusted.

The limited use of accessions preserved in germplasm banks for crossing is one of the limitations of TFG breeding (Valle et al., 2009; Pereira et al., 2018). Other limitations include the use of a large number of candidate genera and species, insufficient information on the biology of the species, low genetic variability for important traits, polyploidy, a complex mode of reproduction (apomixis), wild characteristics of important species (dehiscence and malformation of seeds, anti-quality factors, and sensitivity to photoperiod), lack of information on the genetic control and heritability of agronomic traits, and little participation of the private sector in the development of cultivars (Valle et al., 2009; Sandhu et al., 2015; Pereira et al., 2018). It is worth noting that brachiaria, Guinea grass, and elephant grass are perennial species, which implies that most traits are evaluated in the field over long periods, including several cuts. It is common for a specific trait to show variation among different cuts (Rocha et al., 2019), which is very different compared to that of annual species. The impact of these limitations, along with the differences in market demands and the ability of producers to absorb new releases, can be seen in the low number of cultivars released through the years when compared to the release of grain and fiber crops (Figure 1).

**FIGURE 2:**
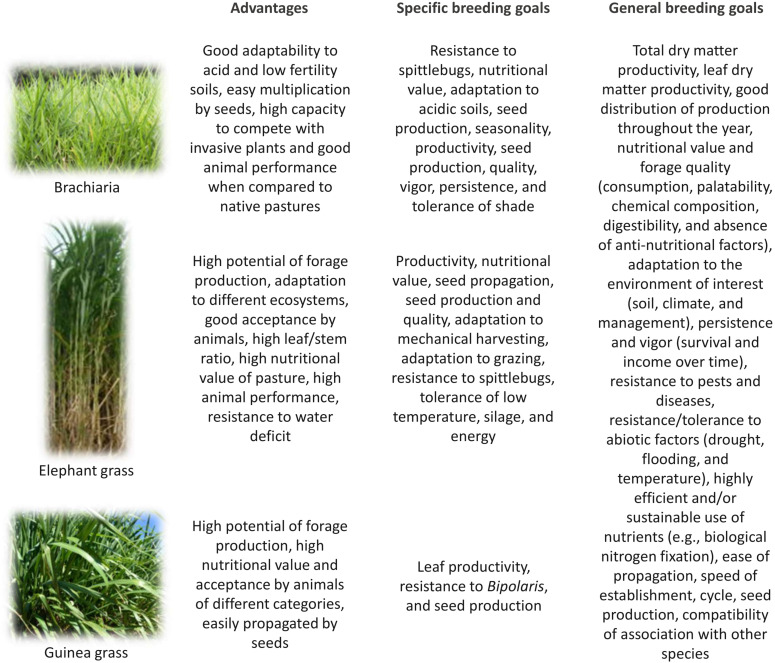
Characteristics of brachiaria, Guinea grass, and elephant grass and breeding goals to improve their use as tropical forage grasses. The advantages and breeding goals are based on Machado et al. (2019). Source of the pictures: Embrapa.

Despite these limitations, the improvement of forage grasses has revolutionized the pastoral systems. In a study conducted in sub-Saharan Africa, legume, and grass cultivars released for different animal production systems increased forage production by 2.65 times when compared to that of traditional cultivars. Production was even higher when only forage grass was used (Paul et al., 2020). Until recently, germplasm introduction was the key method used for forage grass breeding, which involved the evaluation and selection of germplasm accessions as a strategy to obtain cultivars. This method was used to release *U. brizantha* cv. Marandu in 1984 by EMBRAPA (Nunes et al., 1984), which is currently cultivated on fifty-mega hectares of land in Brazil alone (Jank et al., 2014). The germplasm introduction method, albeit simple, rapid, and cost-effective, tends to be prone to exhaustion as it requires the use of accessions collected from nature or accessions obtained from a germplasm bank in other breeding programs (Jank et al., 2011). In addition, natural habitats of species are being increasingly degraded, with loss of variability as well as restrictions in free access to germplasm across different countries and breeding programs, notably due to recent laws for access to genetic diversity and protection of cultivars (Pengelly and Maass, 2019). Since 2000, the use of recombination as a key strategy for cultivar development has intensified. For example, among the new cultivars released by various breeding programs, intra- or interspecific hybrids, especially of *Urochloa*, *Megathyrsus*, and *Cenchrus*, have been highlighted, in which the favorable traits of their progenitors are gathered. Of note, in the last decade, long-term recurrent selection programs have been established for major species, and promising results have been achieved (Miles et al., 2006; Reis et al., 2008; Barrios et al., 2013). The main objectives of TFG breeding are to identify and develop improved genotypes that contribute to increased animal productivity and reduced environmental impact (Figure 2). Thus, not only a better agronomic behavior of the plant, but also a more productive performance of the animal is sought, while ensuring minimal environmental impact (Valle, 2001).

Although, TFG breeding programs have been successful in releasing new and important cultivars over the years, there are certain challenges to overcome. In Brazil, these challenges include reducing losses due to biotic stresses (especially spittlebug attacks), increasing adaptation based on expected climate changes, and improving nutritional value to enhance animal performance, resulting in more beef and milk per kilogram of pasture. To address these challenges, research priorities have focused on the development of new capabilities such as the availability of genome sequences, high-throughput genotyping, and germplasm characterization of tropical forage grasses; identification of genes associated with important traits; development and use of large-scale phenotyping tools; and implementation of GS (Pereira et al., 2018).

Therefore, the prospects of applying genomic tools in TFG breeding programs are promising, and these tools, coupled with adequate pasture management, can continue to promote substantial advances in livestock productivity. For each forage species, the objectives of the breeding programs should be well-defined, as highlighted in Figure 2. In addition to clear objectives, it is important to use the latest technologies available to accelerate the development of cultivars. In this regard, the use of genomic tools for TFG breeding is fundamental.

## GS: An Appraisal in TFG Breeding

### Peculiarities in Breeding Perennial TFGs: Polyploidy and Apomixis

Perennial forages require selection methods that consider the effects of both between families and within family individuals for higher selection gains, mostly in lower magnitudes of narrow-sense heritability (NSH) (Simeão-Resende et al., 2013, 2014). In forage breeding, a combination of GS methods is expected to be useful for predicting genomic breeding values (GEBVs) and total (genotypic) genomic values (GETVs), for clonally propagated cultivars. The estimation of marker effects and genomic values should enable an increase in selection accuracy and may reduce the time required for completing a breeding cycle and the evaluation cost per genotype. Forage breeding methods associated with GS show differential accuracy and gains, as demonstrated by Simeão-Resende et al. (2014).

Considering all these premises, and the methods of the current breeding programs, the uses, and advances of GS in tropical forages will be presented. Firstly, facts about perennial TFGs must be pointed out, as most are polyploids and reproduce apomictically. Apomixis is asexual reproduction by seed (Barcaccia et al., 2020) producing genetically identical progeny (Hand and Koltunow, 2014). In both important genera of TFG, *Megathyrsus* and *Urochloa*, gametophytic apomixis subtype apospory occurs (Ozias-Akins and van Dijk, 2007) which is a mode of reproduction in which the embryo originates from a polyploid nucellar cell as a maternal clone by the seed (Valle and Savidan, 1996). Therefore, the commercial cultivars of these species are generally both polyploid and apomictic.

Autotetraploid individuals have been developed in *U. ruziziensis* and *M. maximus* by artificially duplicated chromosomes from diploid sexual individuals (Jank et al., 2014). These sexual individuals are essential for hybridization with apomictic ones in breeding programs of both genera to increase genetic variability and enhance selection. Therefore, cytogenetic analysis is constantly performed in parents and hybrids and should be evaluated by considering the importance of the species targeted by GS.

According to Bourke et al. (2018), the knowledge of the meiotic behavior of a species is sometimes required to analyze polyploid data using dosage calling software that uses the expected segregation ratios in the F_1_ autotetraploid population. Unlike allotetraploids, autotetraploids do not behave like diploids during meiosis and require specialized methods and tools for genetic studies and mapping (Gallais, 2003). Autotetraploid plants exhibit polysomic inheritance, which can be detected during a cytogenetic analysis by visualization of tetravalent formation and segmental pairing among “partially homologous” chromosomes (Stebbins, 1947) as well as by molecular inference (Worthington et al., 2016). The consequence of chromosome pairing in a tetravalent is the generation of unbalanced gametes and individuals with non-Mendelian inheritance. Even in recent autotetraploids induced by colchicine, chromosome pairing may not show tetravalent formation or other meiotic abnormalities (Pagliarini et al., 2008); however, the four alleles per locus are always present. This may generate errors in genetic mapping, haplotype designation, and the estimation of marker effects, which are important factors in genomic prediction.

Diploid sexual individuals of *M. maximus* were collected in Korogwe, Tanzania, and artificially duplicated (Jank et al., 2014). The cytogenetic evaluation of autotetraploid (2n = 4x = 32) sexual and supposedly segmental allopolyploid apomictic plants revealed a low-to-moderate rate of meiotic abnormalities among sexual (5%–31%) and apomictic (7%–11%) parents (Pessim et al., 2010, 2015). Hybrids originating from a single cross showed abnormal cells at a rate ranging from 16% to 52% (Pessim et al., 2010, 2015). The frequency of meiotic abnormalities found in *M. maximus* is lower than that reported in the tetraploid *Urochloa* (2n = 4x = 36) interspecific hybrids, which ranged from 18% to 82% (Risso-Pascotto et al., 2005; Mendes-Bonato et al., 2006, 2007; Fuzinatto et al., 2007). Pagliarini et al. (2008) found that the mean occurrence of meiotic abnormalities in five induced autotetraploid *U. ruziziensis* accessions ranged from 5% to 10% and only one accession reached 55% abnormalities. However, contrary to expectation, in all the autotetraploidized accessions, chromosome pairing was preferentially bivalent (Pagliarini et al., 2008).

Regardless of the low rate of tetravalent formation in tetraploid and sexual *M. maximus* and *U. ruziziensis*, the issues of allele dosage and compatibility between apomictic and sexual genomes still remains unresolved, as explained by Gallais (2003) for recently doubled genotypes. Therefore, efficient cytogenetic identification of the best crosses at early stages would allow for the identification of the best and most cytogenetically stable parents and progenies. Consequently, all subsequent stages of breeding programs will certainly benefit from genomic prediction and the unbiased estimation of marker effects.

### Availability of TFG Breeding Populations for GS

**FIGURE 3:**
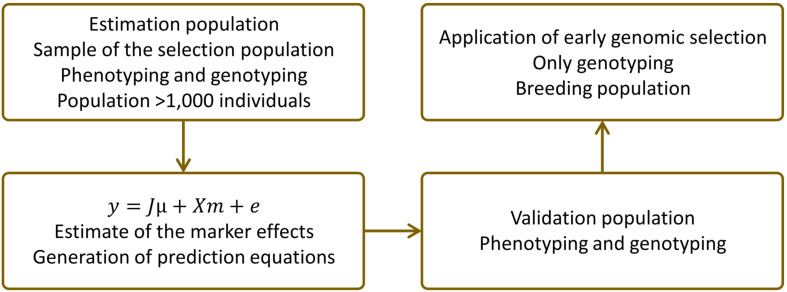
Schematic application of genomic selection (GS) in a genetic improvement program (Resende et al., 2012).

In practice, three populations must be defined for GS: estimation, validation, and breeding populations (Goddard and Hayes, 2007; Meuwissen, 2007). These populations may be as follows: i) physically distinct (three different populations), ii) with two simultaneous functions (only one population used for estimation and validation), or iii) with three simultaneous functions (only one population used for estimation, validation, and selection). Figure 3 illustrates strategy ii.

#### Estimation Population

The estimation population is also called the discovery, training, or reference population. This dataset includes a large number of markers assessed in a moderate number of individuals (1,000 to 2,000 depending on the desired accuracy), which should have their phenotypes assessed for various traits of interest. Equations for predicting genomic values (random multiple regression) are obtained for each trait. These equations associate each marker or interval with its effect (predicted by RR-BLUP) on the trait of interest. The markers that explain the loci regulating the traits are identified in this population, and their effects are estimated. Recently, Lara et al. (2019) and Matias et al. (2019a) used estimation populations with 530 individuals of *M. maximus* and 272 individuals of *Urochloa* hybrids, respectively. Predictive abilities were lower than 0.4 for the evaluated traits. This indicates that factors affecting GS efficiency in TFG breeding, such as adequately sized training populations, still need to be improved.

#### Validation Population

When physically separated from the estimation population, this dataset is smaller than the discovery population and includes individuals that are assessed for SNP markers and various traits of interest. The equations for predicting genomic values are tested to verify their accuracy for this independent sample. To calculate the accuracy, genomic values are predicted, using the estimated effects from the estimation population and subjected to correlation analysis with the observed phenotypic values. As the validation sample is not involved in predicting the marker effects, errors from genomic values and phenotypic values are independent. Correlations between these values are predominantly genetic in nature and equivalent to the predictive ability (r_*yŷ*_) of GS in estimating phenotypes, which is given by the accuracy of the selection itself ($r_{q\overset{\wedge }{q}}$) multiplied by the square root of the heritability (*h*), or $r_{y\,\hat{y}}=r_{q\,\hat{q}}\,h$. Thus, to estimate the accuracy, one should obtain $r_{q\,\hat{q}}=r_{y\,\hat{y}}/h$. This method is valid when raw phenotypic values are used to calculate the correlations. When using genotypic values predicted based on phenotypes instead of raw phenotypic values, heritability should be replaced by the reliability of the prediction. In general, strategy ii is adopted according to a k-fold scheme for cross-validation. According to Meuwissen (2007), when dozens to hundreds of thousands of haplotypes are estimated, there is a risk of over-parameterization; in other words, errors in the data are explained by the marker effects. Cross-validation is therefore extremely important to address this problem.

#### Breeding Population

This dataset only contains the markers assessed in the candidates for selection, and the phenotypes do not need to be assessed in this population. Therefore, the prediction equations derived from the estimation population are used to predict the GVs or future phenotypes of the candidates for selection. The associated selection accuracy is calculated for the validation population.

In most TFG breeding programs, the training population is the same as, or part of, the breeding population, and this population may have experienced directional selection for many generations (Simeão-Resende et al., 2014). It is likely that validation may never exist for most breeding programs, firstly because cross-validation (Kohavi, 1995) has been the commonly used method and secondly because it is difficult to find out more than one ongoing breeding program per species and country.

Elephant grass (*Cenchrus purpureus*) is a tetraploid and allogamous species in which open pollinated divergent populations are easy to establish. The two main breeding strategies are: (i) recurrent selection (Reis et al., 2008); and (ii) clonal selection (Pereira et al., 2017; Machado et al., 2019). The recommended number of individuals in estimation populations for GS can be easily reached, in which the effective population size can be previously assumed and effectively estimated after genotyping. Validation can be performed by cross-validation and this may be extended to different environments or even to related populations of breeding programs in other countries.

Guinea grass (*M. maximus*) and brachiaria (*Urochloa*) breeding programs have recently adopted the full-sib reciprocal recurrent selection as a method, in which thousands of hybrids are generated annually (Barrios et al., 2013; Worthington and Miles, 2015). As the intrapopulation recurrent selection is feasible only on sexual populations, because there is no possible crossing between apomictic accessions, selection is performed on sexual individuals as a function of heterosis expressed in crossings with apomictic accessions. The schematic drawing of this procedure was presented for *M. maximus* (Simeão-Resende et al., 2004) and *Urochloa* (Jank et al., 2014). Based on this information, and the fact that these methods have been recently implemented, some limitations for the establishment of estimation populations in these genera must be discussed. Firstly, the N_*e*_ of the sexual population is extremely low (N_*e*_ <7) in *M. maximus* (Simeão-Resende et al., 2004; Lara et al., 2019), in tetraploid *U. ruziziensis* used as female parents in crossings with apomictic *U. brizantha* (Simeão et al., 2015), and in tetraploid sexual *U. decumbens* (Barrios et al., 2013). Secondly, albeit the genetic diversity within populations of apomictic accessions of *Urochloa* species is high (Vigna et al., 2011), the number of accessions used in crosses is low, because the crosses are performed based only on the adapted and agronomically selected apomictic individuals (Jones et al., 2021). Therefore, the N_*e*_ is likewise low (<20). Thirdly, as a result of the intra or interspecific crosses, F_1_ progeny segregates for mode of reproduction in which individuals in the progeny may vary from 99% apomictic to 99% sexual. While this procedure is efficient to explore the panmictic heterosis (Lamkey and Edwards, 1999) and can readily generate apomictic individuals that are potential cultivar candidates, these hybrid swarms do not constitute a breeding population *per se*. Therefore, the N_*e*_ among and within hybrid families must be adjusted and considered for efficient GS use in both genera. Using the equation $N_{e}=\frac{4\,N_{f}\,n}{n+1},$ in which *N*_*f*_ is the number of full-sib families and *n* the number of individuals per family, we may suggest the evaluation of 42 families and 10 individuals per family, a total of 420 genotyped individuals, to achieve *N*_*e*_ of approximately 152. For phenotyping, the total number may be approximately 1000 individuals (Resende, 2015). Finally, in practice, the TFG estimation population for GS in the genera *Megathyrsus* and *Urochloa* could be composed of sexual hybrids in an open pollinated population, followed by validation in apomictic hybrids.

### Genetic Markers in Selection and Gene Discovery

The use of molecular genetic markers for selection and genetic improvement is based on the genetic linkage between these markers and a quantitative trait locus (QTL) of interest (Resende et al., 2013). Thus, linkage disequilibrium (LD) between markers and QTLs is essential for genomic selection from genomic information (Bourke et al., 2018). It must be made clear that a QTL refers only to the statistical association between a genomic region and a trait.

Recently, molecular genetic markers that consist of SNPs (based on the detection of polymorphisms that arise from a single nucleotide change in the genome) have been widely used in many species (Elshire et al., 2011). Generally, for a SNP to be considered genetically derived, the polymorphism must occur in at least 1% of the population (Resende, 2015). SNPs are the most common type of genomic variation and preferred over other genetic markers because of their abundance, ease of obtainment, and low genotyping cost. Thousands of SNPs can be used to cover the entire genome of an organism with markers not more than 1 cM apart from each other.

LD analysis is based on LD between a marker and a QTL in the whole population and not only within a family, as performed in linkage analysis (Würschum, 2012). For this to occur, the marker and QTL must be closely linked. When this occurs, the association between them is a property of the entire population and persists for many generations.

Association analysis is used for fine mapping and is based on population-level LD (Resende et al., 2013). Linkage can occur when the gene directly affects a trait, and when there is an LD between the marker and the gene controlling the trait. In the first case, the effect of the gene is directly evaluated, and the marker is classified as functional. The functional mutations are known as quantitative trait nucleotides (QTNs). In the second case, the linkage test requires LD between the marker and QTL. When a mutation occurs on a given chromosome, it creates a haplotype with adjacent loci on the chromosome. In the subsequent generations, this mutation tends to occur within the same haplotype unless there is recombination, which creates the LD used for association mapping (Resende et al., 2013).

In recent times, efforts to use molecular markers in genetic improvement research have evolved into two approaches: (1) GWAS for QTL identification and mapping; and (2) genome-wide selection (GWS) or GS (Resende, 2015). GS was proposed by Meuwissen et al. (2001) to increase breeding efficiency and accelerate genetic improvement. GS emphasizes the simultaneous prediction (without the use of significance tests for individual markers) of the genetic effects of thousands of SNP markers dispersed throughout the genome of an organism to capture the effects of all loci (both small and large effects) and identify the overall genetic variation of a quantitative trait (Resende and Alves, 2020). In this case, the sum of the estimated genetic effects of the markers present in an individual provides the genetic value of the individual for selection purposes. Meuwissen et al. (2001) obtained a complete array of estimates of haplotype effects using the ridge regression best linear unbiased prediction (RR-BLUP), BayesA, and BayesB methods. RR had already been used by Whittaker et al. (2000) for marker selection. Haley and Visscher (1998) suggested the name GS for selection on a whole genome scale (Resende and Alves, 2020).

The conceptual development of GS coincides with the technology associated with SNPs, which is accurate and relatively affordable. GS uses the associations between many SNP markers throughout the genome along with phenotypes and takes advantage of LD between markers and QTLs in close linkage (Resende et al., 2013). The predictions derived from phenotypes and SNP genotypes with high density in a generation are thus used to obtain genomic values (GVs) for individuals in any subsequent generations based on their genotypic markers, in which the genetic effects have been estimated.

When LD between markers is incomplete, the joint allele frequencies for the two loci can change markedly across generations, thereby leading to changes in haplotypes. In this case, the marker effects would need to be estimated again to maintain the accuracy of GS for various generations (Dekkers, 2007). In the case of a complete or close LD, the estimated effects remain constant across different families and generations within the same environment.

The number of markers used are directly associated to the genome size, extent of LD and population structure (Ballesta et al., 2020). Larger genomes, rapid breakdown of LD and greater effective population size imply that a higher density of SNP loci would be needed.

### Factors Affecting the Efficiency of GS

The accuracy of GS depends on five factors (Resende et al., 2014): i) heritability of the trait; ii) number of loci regulating the trait (also given by 2N_*e*_L) and the distribution of their effects; iii) number of individuals in the discovery population; iv) effective population size (N_*e*_); and v) marker density, which depends on the number and genome size (L, in Morgans). The first two factors are beyond the breeder’s control, and the latter three factors can be modified by the breeder to increase the accuracy of GS.

An increase in the selection efficiency using GS can be achieved by changing the four components of the expression for genetic progress, given by $S\,G=\left(k\,r_{g\,\hat{g}}\,\sigma_{g}\right)/T$, where *k* is the standardized selection differential (dependent on the selection intensity), $r_{g\overset{\wedge }{g}}$ is the accuracy of selection, σ_*g*_ is the genetic standard deviation (genetic variability) of the trait in the population, and *T* is the time required to complete a selection cycle.

In perennial and vegetatively propagated plant species, the benefit of GS is from an increase in $r_{g\overset{\wedge }{g}}$ and a reduction in *T*. The increase in $r_{g\overset{\wedge }{g}}$ is due to the use of an actual kinship matrix (Resende, 2007). This increase depends on the size of the estimation population and marker density. Factor *T* is greatly reduced by GS because genomic prediction and selection can be performed at the seedling stage. Thus, even if $r_{g\overset{\wedge }{g}}$ shows the same magnitude as obtained by phenotypic selection, GS is still better than selection based on phenotypes due to the reduction of *T*.

### Inferring the Quality and Efficiency of GS

The quality of GS is inferred by correlation and regression among the predicted genetic values and phenotypes in the validation population, as well as by the accuracy of the prediction. The correlation and regression coefficients involving observed and predicted values are practical measures of the ability of the methods to make predictions that are accurate and unbiased, respectively. Correlation provides predictive ability, which is equivalent to the product of accuracy and the square root of heritability (Resende et al., 2014). The regression coefficient is algebraically equal to 1. Regression coefficients of less than 1 indicate that the genetic values are overestimated and exhibit greater variability than expected; coefficients greater than 1 indicate that the estimated genetic values exhibit variability lower than expected. A lack of bias is important when selection involves individuals from many generations using the estimated marker effects from a single generation. Regression coefficients near 1 indicate that the assessments are unbiased and effectively predict the actual magnitudes of differences among the individuals assessed.

The expected value of the regression coefficient is 1, which indicates an unbiased prediction. Thus, the regression coefficient can also be used to estimate the heritability of the markers (Resende et al., 2013). Various heritability values are assessed, and those that provide a regression equal to 1 should be selected as the best estimate. If the regression yields a result less than 1, the magnitude of the assessed heritability value is too high and should be reduced until the regression coefficient is converged to 1. If the regression yields a result greater than 1, the magnitude of the assessed heritability value is too low and should be increased until it converges to 1.

## Current Genomic Resources for TFG Breeding

The availability of a large number of high-quality single-nucleotide polymorphisms (SNPs) that can be genotyped at a reasonable cost is a prerequisite for implementing GS in a crop of choice (Hayes et al., 2013). Gold-standard methods for SNP discovery rely on resequencing individual samples at minimum coverage and mapping reads to a reference genome (McKenna et al., 2010); this is also true for low-coverage, genotyping-by-sequencing (GBS) methods (Elshire et al., 2011). Although, reference-free pipelines are available for GBS, such as UNEAK (Lu et al., 2013), reference-based methods allow for more informed decisions during the selection of high-quality SNPs (McCormick et al., 2015). Alternative methods have been recently tested and reported in *Urochloa*, for which the GBS-SNP-CROP pipeline (Melo et al., 2016) was used to generate a “mock” reference from GBS data (Matias et al., 2019b). This led to the discovery of a larger number of biallelic SNPs, when compared to mapping reads to the available genome of the closest related species (*Setaria viridis* and *Setaria italica*).

Assembly quality, which is measured by its accuracy, haplotype phasing, and contiguity, is an important factor influencing marker discovery, GWAS, and GS. In tetraploid blueberries, the selection of probes for targeted SNP genotyping, investigation of the genetic architecture of fruit traits, identification of candidate genes, and genomic prediction benefited from a more complete, chromosome-scale, haplotype-phased genome assembly (Benevenuto et al., 2019). A huge reduction in sequencing costs and increased throughput brought about by second-generation sequencing have allowed unprecedented access to crop genomic information (Shamshad and Sharma, 2018). However, the genomic complexity of most TFG species is still challenging and has kept them recalcitrant to sequencing efforts targeting reference-grade assemblies using mainly second-generation short reads. Forage grass species with the largest breeding programs in Latin America are mostly polyploid and highly heterozygous and have genomes with a high repeat content (Table 1). Third-generation long reads can circumvent some of these problems, and, combining with a technology such as optical mapping or chromosome conformation capture can potentially allow chromosome-scale reference-grade assemblies (Belser et al., 2018; Shi et al., 2019). The use of these tools to accelerate genomic research on TFGs is promising and can thus advance the use of GS in breeding programs. We argue that the availability of high quality genome assemblies is the starting point for the development of genotyping systems that will be useful for successfully deploying GS in TFG breeding programs.

**TABLE 1:** Reproductive system and genomic information of economically important tropical forage grasses used in livestock production.

Scientific name	Predominant reproductive system	Genome size (Gpb)	Chromosome number and ploidy level	WGS^#^
*Urochloa brizantha*	Apomictic	1.4	2n = 4x = 36	No
*Urochloa humidicola*	Apomictic	1.9	2n = 6x, 9x = 36 to 54	No
*Urochloa decumbens*	Apomictic	1.6	2n = 4x = 36	No
*Urochloa ruziziensis*	Sexual	0.6	2n = 2x = 18	Yes
*Megathyrsus maximus*	Apomictic	1.0	2n = 4x = 32	No
*Cenchrus purpureus*	Sexual	2.1	2n = 4x = 28	Yes

*^#^WGS, availability of whole-genome sequence.*

### Genome Assembly in *Urochloa* spp.

*Urochloa* P. Beauv. Grasses, many of which were previously included in *Brachiaria* (Trin.) Griseb., are the most widely used forage in Latin America. The main species are *U. ruziziensis*, *U. brizantha*, *U. decumbens*, and *U. humidicola*. The first genome assembly of a forage grass species in the genus *Urochloa* has recently been reported for *U. ruziziensis* (Worthington et al., 2020). The diploid genotype CIAT26162 was sequenced using short reads (approximately 100 × coverage). Assembly and scaffolding were performed and PacBio RSII reads were used for gap filling. This is an invaluable resource for an orphan species with no previously published genome information available. However, it also highlights the huge challenges in the assembly of highly heterozygous, repetitive genomes with short-read technologies. The publicly available assembly was fragmented into 102,577 scaffolds, with an N50 of 27.8 kbp. Completeness metrics based on benchmarking universal single-copy orthologs (BUSCOs) indicate that 86.7% are complete (1,248 out of 1,440 in the Embryophyta_odb9 dataset), suggesting that there is still room for improvement. Because of the lack of linkage maps for an intraspecific diploid cross in *U. ruziziensis*, which could be used to cluster and order scaffolds based on linkage groups, the study relied on anchoring scaffolds based on synteny with the *Setaria italica* genome to obtain pseudo-molecules. This potentially affected the anchored assembly due to undetected chromosomal rearrangements that might be present between *S. italica* and *U. ruziziensis*, as phylogenetic information and evolutionary relationships were not taken into account in the anchoring process.

### Genomic Resources for Guinea Grass (*M. maximus*)

The placement of Guinea grass in the phylogeny of Paniceae has changed over the years, however, plant breeders, seed producers, and farmers in Latin America mostly refer to it as *Panicum maximum*, or “Panicum” as a common name for the species. There is no reference genome assembly available for *M. maximus*. The fact that Guinea grass was once included in *Panicum* and is still mostly regarded as a *Panicum* species in Latin America may lead to the assumption that the available genome assemblies for panic grasses such as *P. hallii* (two publicly available chromosome-scale assemblies; Lovell et al., 2018) and *P. milliaceum* (two chromosome-scale assemblies; Shi et al., 2019; Zou et al., 2019) would provide suitable shortcuts for the development of genomic resources for *M. maximus*. Lara et al. (2019) illustrated how this approach might limit the genomic distribution and number of SNPs available when genotyping *M. maximus* breeding populations using *Panicum* genome assemblies as references. In this study, a multi-parental population of *M. maximus* half-sib progenies was genotyped using GBS, and six different assemblies were tested as references for read mapping. Two of them comprised the genome assemblies for *P. hallii* and *P. virgatum*, while the remaining included genome assemblies for *Setaria* (*S. italica* and *S. viridis*) and transcriptome assemblies for *M. maximus*. The alignment rates ranged between 19.05% for *P. hallii* and 24.24% for *M. maximus* transcriptomes, showing that a very large proportion of reads were not used for SNP discovery and genotyping. The sets of allele-dosed SNPs containing up to 5% of missing data from each of the reference assemblies ranged between 5,032 for one of the *M. maximus* transcriptomes and 8,112 for *S. viridis*. Although, this was the first report on the development and assessment of prediction models, considering the allele dosage, for GS in *M. maximus*, increased marker density and prediction accuracies may be expected when a high-quality genome assembly for the species is available for SNP discovery and genotyping.

### Genomic Resources and Genome Assemblies for Elephant Grass (*C. purpureus*)

Elephant grass (*C. purpureus* Schumach. Morrone, syn. *Pennisetum purpureum* Schumach.), also called napier grass, merker grass, or Uganda grass, is a tropical grass native to Eastern and Central Africa. It is an allotetraploid species with a chromosome constitution of 2n = 4x = 28 A’A’BB and an average amount of DNA per G1 nucleus of 4.58 pg (Hanna, 1981; Taylor and Vasil, 1987). Elephant grass is used as animal fodder and is a promising lignocellulosic biofuel feedstock due to its high growth rate, high biomass yield, and persistence (Morais et al., 2009; Singh et al., 2013; Daud et al., 2014; Rocha et al., 2019).

Wang et al. (2018) conducted a genome survey of elephant grass and estimated its genome size to be 2.01 Gb with 71.36% of repetitive elements and a heterozygosity of 1.02%. A total of 114.36 Gb of raw data, (approximately 57-fold coverage) was generated using the Illumina HiSeq sequencing platform for the Zise genotype (purple elephant grass). A partial draft assembly was obtained using SOAPdenovo. As expected for such a complex genome, this effort allowed a preliminary investigation of the repetitive content of elephant grass and the identification of thousands of genomic SSR markers, 30 of which were tested for genotyping a set of 28 elephant grass accessions. Another genome survey of Merkeron and UF1 cultivars was conducted by Paudel et al. (2018), and they also developed a high-density linkage map using GBS.

More recently, two chromosome-scale assemblies of elephant grass were reported (Yan et al., 2020; Zhang et al., 2020). The initial assembly of the cv. Purple (Yan et al., 2020) was obtained using Nanopore long reads and was then polished with Illumina short reads and scaffolded with Hi-C data. Approximately 2,000 contigs were grouped and oriented into 14 chromosome-scale scaffolds, with a total size of 1.9 Gbp, 66.3% of which were annotated as repetitive elements. The assembly showed high contiguity at the contig level (N50 1.8 Mbp) and 97.8% completeness using BUSCOs. The predicted protein-coding gene set also showed high BUSCO completeness (97.1%).

The second assembly (Zhang et al., 2020, available as a preprint), for the CIAT6263 accession, was obtained with Nanopore reads and ultra-long reads, which were assembled to a total size of 2.07 Gbp with a contig N50 of approximately 2.9 Mbp. The authors used a combination of BioNano optical maps and Hi-C to obtain a final chromosome-scale assembly of approximately 2 Gbp in 14 pseudomolecules. This assembly was also 97.8% BUSCO complete, and repetitive elements accounted for 60.7% of the genome. The difference in repetitive content between the two assemblies might be explained by the different methods applied for *de novo* identification of repeats.

These two assemblies place elephant grass in a unique position among the TFGs. Current research trends indicate the benefits of having not only one but multiple genome assemblies for a species of interest (Della Coletta et al., 2021). Large sequencing projects now target pan-genomes, instead of a single reference that does not capture the full diversity of a species (Zhao et al., 2018). Future efforts of variant discovery and association of phenotypes with genomic locations will be possible using these assemblies as anchors for read mapping, opening up the possibility of revisiting previous datasets that were generated without a reference genome. However, while both efforts resulted in chromosome-scale scaffolds, the fact that the SMARTDENOVO assembler does not generate haplotype-resolved contigs indicates that the high heterozygosity levels of elephant grass were not represented in the assemblies.

## Statistical Methods in GS

An ideal method for estimation of SNP effects in GS should accommodate the genetic architecture of the trait in terms of genes of small and large effects and their distributions, regularize the estimation process in the presence of multicollinearity and a larger number of markers than individuals, using shrinkage estimators, and perform the selection of covariables (markers) that affect the trait under analysis. The main problem with GS is the estimation of a large number of effects from a limited number of observations and the collinearities arising from LD between the markers. Shrinkage estimators deal with this appropriately by treating the effects of markers as random variables and estimating them simultaneously (Resende, 2007; Resende et al., 2008; Azevedo et al., 2015; Resende and Alves, 2020).

If the effects of markers are taken as fixed, it is not possible to consider the covariance between the effects of the markers. With a high density of markers, more than one marker will be in LD with a segregating QTL, which will result in covariance between the marker effects. Most markers will have no effect on a trait, and the estimated effects of these empty markers will be false. This problem is greater when the markers are considered to have fixed effects, because in that case, these pseudo effects will not shrink toward zero (Resende and Alves, 2020).

In the context of marker-assisted selection (MAS) and genomic prediction, the method of least squares (LS) has serious drawbacks. According to Gianola et al. (2003), the selection index (calculated as the regression involving molecular scores) presented by Lande and Thompson (1990) for MAS fails when formulated vectorially. This failure occurs because the covariance matrix for the molecular scores is singular, as the distribution of fitted regression values is defined only in the p-dimensional space (number of covariables) and not in the n-dimensional space (number of individuals with molecular scores). Therefore, the selection index leads to an infinite number of solutions.

Another difficulty arises when the number of markers is equal to or greater than the number of genotyped individuals. In this case, the collinearity of the predictor variables causes parametric identification problems, and thus, some type of dimensional reduction, such as singular value decomposition, should be used. Another problem is the inadmissibility (unable to provide the minimum mean square error) of LS estimators, a result that collapses estimates by LS and generalized LS (GLS). Thus, the LS method is not recommended for the MAS and GS analyses. In summary, the LS method is inefficient because it is impossible to simultaneously estimate all effects when the number of effects to be estimated is greater than the number of data points; thus, estimating one effect at a time and testing its significance leads to an overestimation of significant effects, and the accuracy of the method becomes low. In addition, only QTLs with large effects will be detected and used, and consequently, not all genetic variations will be captured by the markers. The LS method assumes *a priori* QTL distribution, with an infinitely large variance that disagrees with the known total genetic variance.

Because the number of markers in GS is greater than the number of individuals, there is a lack of degrees of freedom to estimate the effects of all markers. A solution to this problem is to use the RR method (Whittaker et al., 2000) or to consider the marker effects as random instead of fixed. Fitting random effects does not expend degrees of freedom, and the effects of all markers can be estimated simultaneously. This method leads to RR-BLUP, which considers the effects of QTLs with normal distributions and equal variance through chromosomal segments.

The main problem for GS is estimating a large number of effects from a limited number of observations, in addition to collinearities resulting from LD between markers. The shrinkage estimators adequately address this issue by treating the marker effects as random variables and estimating them simultaneously (Resende et al., 2008).

The main methods for GS are based on Random Regression and can be divided into three major classes: explicit, implicit, and dimensionally reduced regression. In the first class, the RR-BLUP, Lasso, BayesA, and BayesB methods stand out among others. In the class of implicit regression, the Reproducing Kernel Hilbert Spaces (RKHS) method, which is semiparametric, is the most popular. The Independent Components, Partial Least Squares, and Principal Components stand out among the regression methods with dimensional reduction. Two new non-parametric approaches for GS proposed by Resende (2015) and Lima et al. (2019a,b) have proven to be efficient (Resende and Alves, 2020) and are called triple categorical regression (TCR) and Delta-p, respectively.

The explicit regression methods are divided into two groups: (i) penalized estimation methods (RR-BLUP, Lasso) and (ii) Bayesian estimation methods (including BayesA, BayesB, fast BayesB, BayesCπ, BayesDπ, Bayesian regression, BayesRR, BayesRS, BLasso, and IBLasso). Among these, the best and most effective in practice are RR-BLUP and BayesB (Visscher et al., 2006, 2008, 2010; Mrode et al., 2010; Mrode, 2014). Each method without covariate selection has a similar method with covariate selection. Thus, the following are the pairs without - with covariate selection: BayesA - BayesB; BayesRR - BayesCπ; BLasso - IBLasso (Resende and Alves, 2020).

The RR-BLUP is a model equivalent to genomic best linear unbiased prediction (G-BLUP), which is the BLUP method at an individual level with the genealogical relationship matrix A changed to a genomic relationship matrix G. The equivalence between these two methods was given by Habier et al. (2007) and Van Raden (2008). The G-BLUP and RR-BLUP are equivalent when the number of QTLs is large, and no major QTL is present. The use of matrix G based on markers had already been established by Bernardo (1994); Nejati-Javaremi et al. (1997), and Fernando (1998). A single-step BLUP simultaneously using phenotypic, genotypic, and genealogical information, called H-BLUP single-step, was proposed by Misztal et al. (2009), using an H matrix composed of the A and G matrices (Resende and Alves, 2020). The idea of H-BLUP was given by Fernando (1998).

The traditional quantitative genetics rely on random mating populations. Nowadays, with the availability of SNP markers, random mating does not need to be assumed, because breeders can track the transmission of chromosomal segments. Another assumption is linkage equilibrium in the breeding population. Once linkage among markers is accounted for in the G coefficient matrix in RR-BLUP, this circumvents the need to assume linkage equilibrium (Resende and Alves, 2020).

A refinement of GS can be achieved by using QTNs instead of SNPs. The evolution of genomic technology is predictable and the causal mutation of a genetic variation at the nucleotide level (QTN) can be accessed soon. Thus, GS can be improved by the direct use of QTNs instead of SNPs. The use of QTNs will bring the following advantages (Weller, 2016): GS will not depend on the LD as the QTN will be accessed directly and not via markers and, this will increase the robustness of the genomic prediction, which will also be useful in the long run; the genomic prediction may have transferability across different populations and species in the same genus; genomic prediction will use specific QTNs for each trait, unlike G-BLUP by means of SNPs, which uses the same G relationship matrix for all traits; the multiple-trait selection indices will directly weigh the QTNs and not the phenotypic traits; GS may use a smaller number of generations (only the last ones) for the composition of the G matrix, which will bring greater genetic gain and lesser mass of data to be processed; the allele frequencies of the QTNs will be accessed directly and not through LD with SNPs (Resende and Alves, 2020).

### Single-Environment RR-BLUP and G-BLUP Models

The parametric regression model for a single environment *j*^*th*^ (*j = 1, …, m*) is defined as *y*_*j*_ = 1_*nj*_μ_*j*_ + *X*_*j*_β_*j*_ + ε_*j*_, where the vector *y_j_* represents *nj* independent centered observations of the response variable in the *j*^*th*^ environment; *1*_*nj*_ is a vector of ones of order *nj*; μ_*j*_ is the overall mean of the *j*^*th*^ environment; *X_j_* is the matrix for the *p* centered and standardized molecular markers in the *j*^*th*^ environment; vector β_*j*_ represents the effect of each of the *p* markers in the *j*^*th*^ environment, and ε_*j*_ is the vector of random errors in the *j*^*th*^ environment with normal distribution and common variance $\sigma_{\varepsilon_{j}}^{2}$. The RR-BLUP assumes that the effects of the markers have a multivariate normal distribution $\beta_{j}∼N\,\left(0,I\,\sigma_{\beta {}_{j}}^{2}\right)$.

Assuming that the effects of the markers β_*j*_ and ε_*j*_ are independent, and that *u*_*j*_ = *X*_*j*_β_*j*_, then the above model for the *j*^*th*^ environment can be written as *y*_*j*_ = 1_*nj*_μ_*j*_ + *u*_*j*_ + ε_*j*_, where *u_j_*, and ε_*j*_ are independent random variables with $u_{j}∼N\,\left(0,\sigma_{u_{j}}^{2}\,K_{j}\right)$, and $\varepsilon_{j}∼N\,\left(0,\sigma_{\varepsilon }^{2}\,I\right)$, respectively; $\sigma_{u_{j}}^{2}$ is the variance of *u_j_* (to be estimated), and *K_j_* is a symmetric matrix representing the covariance of the genetic values. Thus, for a single-environment where the *K_j_* is of the linear form $K_{j}=G_{j}=X_{j}\,X_{j}^{\prime }/p$ the G-BLUP is equivalent to RR-BLUP (Van Raden, 2008).

### Genetic Parameterization of Additive, Dominance, and Total Genotype Effects

#### Additive Model

The following linear mixed model can be fitted to estimate the marker effects *y* = *J*μ+*Xm*+*e*, where *y* is the vector of phenotypic observations, *μ* is the vector of the fixed effect of the general mean, *m* is the vector of random marker effects and *e* is the vector of random residuals. *J* and *X* are the incidence matrices for *μ* and *m*, respectively. The incidence matrix *X* contains functions of the values 0, 1, and 2 for the number of alleles for the marker (or the supposed QTL) in a diploid individual. A similar coding method uses the values of -1, 0, and 1. The genomic mixed-model equations for predicting *m* using the RR-BLUP method are equivalent to $\left[\begin{array}{cc} J^{\prime }\,J & J^{\prime }\,X\\ X^{\prime }\,J & X^{\prime }\,X+I\,\frac{\sigma_{e}^{2}}{\left(\sigma_{a}^{2}/n_{Q}\right)} \end{array}\right]\,\left[\begin{array}{c} \hat{\mu }\\ \hat{m} \end{array}\right]=\left[\begin{array}{c} J^{\prime }\,y\\ X^{\prime }\,y \end{array}\right]$. The total additive genomic value for an individual *j* is given by $A\,G\,V_{j}={\hat{y}}_{j}=\sum_{i}X_{i}\,{\hat{m}}_{i}$, where *X*_*i*_ is equal to 0, 1, or 2 for the genotypes mm, Mm, and MM, respectively, for biallelic and co-dominant markers, such as SNPs.

These prediction equations assume *a priori* that all loci explain equal amounts of genetic variation. Thus, the genetic variation explained by each locus is given by $\sigma_{a}^{2}/n_{Q}$, where $\sigma_{a}^{2}$ is the total genetic variation and *n_Q_* is the number of loci (when each locus is perfectly marked by a single marker), which can be given by $n_{Q}=2\,\sum_{i}^{n}p_{i}\,\left(1-p_{i}\right)$, where *p_i_* is the frequency of the allele of the type M in locus *i*. The genetic variation $\sigma_{a}^{2}$ can be estimated by restricted maximum likelihood (REML) on the phenotypic data in a traditional manner or by the variation among markers or QTL chromosomal segments.

There is no need to use the kinship matrix with the RR-BLUP method. The pedigree-based kinship matrix used for traditional BLUP was replaced by a kinship matrix estimated by the markers. This kinship matrix is a function of *X’X* present in the equations of the mixed model described above. This procedure is more efficient because it effectively captures the kinship produced for each individual and not an average kinship matrix associated with the pedigree.

The parameterization of the incidence matrix *X* uses the values 0, 1, and 2 for the number of alleles of a marker (or supposed QTL) in a diploid individual and 2*p* for individuals with missing marker data. These values should be centered around 0 so that the effects of co-dominant markers are effects of allelic substitution with a mean of 0 in the population. In this case, assuming Hardy-Weinberg equilibrium, the additive genetic variation of the trait in the population is equal to $\sigma_{a}^{2}=2\,\sum_{i}^{n}p_{i}\,\left(1-p_{i}\right)\,\sigma_{m}^{2}$. Thus, the values of *X_i_* should be replaced by 0 – 2*p*, 1 – 2*p*, and 2 – 2*p*, to obtain a variable with a mean of 0. Thus, with centralization, $n_{Q}=2\,\sum_{i}^{n}p_{i}\,\left(1-p_{i}\right)$, should be used for the RR-BLUP method, and the additive genetic effects of individuals are given by $\hat{a}=X\,\hat{m}$.

Additionally, the data for markers in matrix *X* can be standardized as follows for each matrix element *X_i_* corresponding to locus *i*:

*X*_*i*_ = (0 – 2*p*_*i*_)/(Var(*X*_*i*_))^1/2^ if the individual is homozygous for the first allele (mm).
*X*_*i*_ = (1 – 2*p*_*i*_)/(Var(*X*_*i*_))^1/2^ if the individual is hetero- zygous (Mm).
*X*_*i*_ = (2 – 2*p*_*i*_)/(Var(*X*_*i*_))^1/2^ if the individual is homozygous for the second allele (MM).
*X*_*i*_ = 0 if the individual has missing marker data. The quantity *p*_*i*_ is the frequency of the second marker allele.

The cut-off point for including a marker in the analysis can be determined by the minor allele frequency (MAF), which is a measure related to the variation of alleles in the population, given by *MAF* = (1/2*N*)^1/2^ which comes from the standard deviation of a proportion, given by (*pq*)^1/2^/(2*N*)^1/2^, where *N* is the number of genotyped individuals, meaning that the lower the *N* value, the greater the MAF needs to be for accurate estimation of the marker effect (Resende, 2015; Resende and Alves, 2020).

#### Coding and Additive Kinship Matrix in Polyploids

The incidence matrix *X* contains the values 0, 1, 2, 3, and 4 for the number of alleles for the marker (or the supposed QTL) in a tetraploid individual. Analysis by G-BLUP uses the kinship matrix given by $G=\frac{\left(X^{*}\,X^{{*}^{\prime }}\right)}{{\left[2\,\sum_{i}^{n}p_{i}\,\left(1-p_{i}\right)\right]}^{1/2}}$, where X^∗^ is the X matrix after centralization.

#### Additive-Dominance Model

According to the marker model *y* = *J*μ + *W*α + *S*δ + *e*, (where coefficients of α and δ are the additive and dominance effects, respectively), the most appropriate parameterization to estimate the effects on the additive-dominance model (Vitezica et al., 2013; Azevedo et al., 2015) is:

Additive effects (W):

$$W=\left\{ \begin{array}{c} I\,f\,M\,M;2\to 2-2\,p=2\,q\\ I\,f\,M\,m;1\to 1-2\,p=q-p\\ I\,f\,m\,m;0\to 0-2\,p=-2\,p \end{array}\right..$$

The values of W must be centered at zero so that the effects of the codominant markers are effects of allelic substitution (α) with a mean of 0 in the population.

Dominance effects (S):

$$S=\left\{ \begin{array}{c} I\,f\,M\,M;0\to -2\,q^{2}\\ I\,f\,M\,m;1\to 2\,p\,q\\ I\,f\,m\,m;0\to -2\,p^{2} \end{array}\right..$$

#### G-BLUP for the Additive-Dominance Model

The individual mixed model is given by *y* = *J*μ + *Za* + *Zd* + *e*, where *a* is the additive genetic vector of the individuals, and *d* is the dominance genetic vector of the individuals; $a∼N\,\left(0,G_{a}\,\sigma_{a}^{2}\right)$, $d∼N\,\left(0,G_{d}\,\sigma_{d}^{2}\right)$, and e∼N(0,Iσ)e2.

The mixed-model equations for the additive-dominance model are equivalent to $\left[\begin{array}{ccc} J^{\prime }\,J & J^{\prime }\,Z & J^{\prime }\,Z\\ Z^{\prime }\,J & Z^{\prime }\,Z+G_{a}^{-1}\,\frac{\sigma_{e}^{2}}{\sigma_{a}^{2}} & Z^{\prime }\,Z\\ Z^{\prime }\,J & Z^{\prime }\,Z & Z^{\prime }\,Z+G_{d}^{-1}\,\frac{\sigma_{e}^{2}}{\sigma_{d}^{2}} \end{array}\right]\,\left[\begin{array}{c} \hat{\mu }\\ \hat{a}\\ \hat{d} \end{array}\right]=\left[\begin{array}{c} J^{\prime }\,y\\ Z^{\prime }\,y\\ Z^{\prime }\,y \end{array}\right]$, where $G_{a}=\frac{W\,W^{\prime }}{\sum_{i=1}^{n}\left(2\,p_{i}\,q_{i}\right)}$, and $G_{d}=\frac{S\,S^{\prime }}{\sum_{i=1}^{n}{\left(2\,p_{i}\,q_{i}\right)}^{2}}$; *p_i_* and *q_i_* are the allelic frequencies; $\sigma_{a}^{2}=\sum_{i=1}^{n}\left[2\,p_{i}\,\left(1-p_{i}\right)\right]\,\sigma_{\alpha }^{2}$, and $\sigma_{d}^{2}=\sum_{i=1}^{n}{\left[2\,p_{i}\,\left(1-p_{i}\right)\right]}^{2}\,\sigma_{\delta }^{2}$; and $\sigma_{a}^{2}$ and $\sigma_{d}^{2}$ are the additive and dominance genetic variances, respectively.

Adjusting an individual genomic model is equivalent to adjusting an individual traditional model but with the pedigree-based matrices A and D replaced by the genomic kinship matrices *G_a_* and *G_d_* for additive and dominance effects, respectively.

#### H-BLUP and Single-Step BLUP

In a simultaneous analysis of genotyped and non-genotyped individuals via G-BLUP, for a global evaluation of the three classes of individuals in a single step, the same additive model *y* = *J*μ + *Za* + *e* can be fitted with one alteration (replacing matrix *G* with matrix *H*) to the mixed-model equations, according to Misztal et al. (2009)$\left[\begin{array}{cc} J^{\prime }\,J & J^{\prime }\,Z\\ Z^{\prime }\,J & Z^{\prime }\,Z+H^{-1}\,\frac{\sigma_{e}^{2}}{\sigma_{a}^{2}} \end{array}\right]\,\left[\begin{array}{c} \hat{\mu }\\ \hat{a} \end{array}\right]=\left[\begin{array}{c} J^{\prime }\,y\\ Z^{\prime }\,y \end{array}\right]$.

Matrix *H* includes both the relationships, based on pedigree (*A*) and differences between those and the genomic relationships (*A*_δ_), such that *H* = *A* + *A*_δ_. Thus, *H* is given by

$H=\left[\begin{array}{cc} A_{11} & A_{12}\\ A_{21} & G \end{array}\right]=A+\left[\begin{array}{cc} 0 & 0\\ 0 & G-A_{22} \end{array}\right]$, where the subscripts 1 and 2 represent non-genotyped and genotyped individuals, respectively.

The inverse of *H*, which allows simpler calculations, is given by $H^{-1}=A^{-1}+\left[\begin{array}{cc} 0 & 0\\ 0 & G^{-1}-A_{22}^{-1} \end{array}\right]=\left[\begin{array}{cc} A^{11} & A^{12}\\ A^{21} & G^{-1}+A^{22}-A_{22}^{-1} \end{array}\right]$, where $A_{22}^{-1}$ is the inverse of the kinship matrix based on pedigree for only genotyped individuals.

From the estimation of genetic values ($\hat{a}$) by G-BLUP, the estimated marker effects ($\hat{m}$) can be obtained by: $\hat{m}={\left(X^{\prime }\,X\right)}^{-1}\,X^{\prime }\,\hat{a}$. Models with dominance effects (*d*) can also be fitted.

Another important application of this analysis is the estimation of total heritability explained by all the markers simultaneously. With the kinship matrix given by $G=\left(X\,X^{\prime }\right)/\left[2\,\sum_{i}^{n}p_{i}\,\left(1-p_{i}\right)\right]$, total heritability can be estimated by REML using the mixed-model equations to estimate the variance components $\sigma_{a}^{2}$ and $\sigma_{e}^{2}$. The elements of matrix *G* represent the average multilocus kinship and are given by $G_{j\,k}=\left(\frac{1}{n}\right)\,\sum_{i=1}^{n}\frac{\left(x_{i\,j}-2\,p_{i}\right)\,\left(x_{i\,k}-2\,p_{i}\right)}{2\,p_{i}\,\left(1-p_{i}\right)}$. Another favorable feature of G-BLUP is the possibility of directly estimating (by prediction error variance (PEV)) the accuracy of GS. For individuals with known phenotypes, this accuracy is valid for the estimation population without cross-validation. In G-BLUP, the phenotypes of the validation population are replaced by missing data. Therefore, individuals from this validation population will have a validated accuracy estimate.

Models at the level of individuals, including genotype × environment (*ae*) interactions, can also be fitted if there are related individuals within the same environment and across environments. In this case, the model is equal to *y* = *Wb* + *Za* + *Zae* + *e*, where *ae* is the vector of effects from the interaction between additive genetic effects and environmental effects (random), and *Z* is the incidence matrix for *a* and *ae*. The mixed-model equations for predicting *a* and *ae* using the BLUP method are $\left[\begin{array}{ccc} W^{\prime }\,W & W^{\prime }\,Z & W^{\prime }\,Z\\ Z^{\prime }\,W & Z^{\prime }\,Z+G_{a}^{-1}\,\frac{\sigma_{e}^{2}}{\sigma_{a}^{2}} & Z^{\prime }\,Z\\ Z^{\prime }\,W & Z^{\prime }\,Z & Z^{\prime }\,Z+G_{d}^{-1}\,\frac{\sigma_{e}^{2}}{\sigma_{a\,e}^{2}} \end{array}\right]\,\left[\begin{array}{c} \hat{b}\\ \hat{a}\\ \hat{a\,e} \end{array}\right]=\left[\begin{array}{c} W^{\prime }\,y\\ Z^{\prime }\,y\\ Z^{\prime }\,y \end{array}\right]$, where *G*_*ae*_ = *G* for pairs of individuals in the same environment, and *G*_*ae*_ = 0 for pairs of individuals in different environments. The variance of the interaction between the additive genetic and environmental effects is denoted by $\sigma {}_{a\,e}^{2}$.

#### Additive, Dominance, and Total Genotype Effects in Polyploids

As SNP markers are biallelic, the inference of dosage allelic effect is dependent on the genetic effects of interactions (Gallais, 2003; Mackay et al., 2019). Exclusive additivity may create more classes of genotypic values than any other first-degree interaction among alleles and must be studied using allele dosage in GS. In autotetraploids or populations derived from their “allotetraploids,” such as those evidenced in *M. maximus* and *U. ruziziensis*, the additive genetic variance and NSH cannot be estimated based solely on testing half-sib progenies or regression of offspring on the progenitors, because half-sib families may have fractions of the dominant genetic variance (Gallais, 1989). G-BLUP analysis of half-sib polyploid data allows the estimation of broad-sense heritability (BSH) using the information of all genetic relationships available in the kinship marker matrix since some “identity-by-state” dominance relationships allow the estimation of dominance effects, which along with estimated additive genetic effects, provide the estimation of the total genotypic value and then the BSH. This procedure was used in *M. maximus* hybrids (Lara et al., 2019) using a low number of parents and in *Urochloa* interspecific hybrids (Matias et al., 2019a).

Experimental crossing that provides simultaneous half-sib and full-sib progenies should be preferentially designed to estimate additive and dominance genetic effects simultaneously (Simeão-Resende et al., 2004), aiming at the total genotypic-genomic value prediction. In this case, there is more information about dominance relationships, thus generating better estimates. The BSH is estimated by the additive-dominance model, in which g = a + d and var(g) = var(a) + var(d). Making estimations of GEBV and GETV simultaneously in full-sibs and half-sibs with some progenitors in common in a training/validation population will allow the summation of the family effect in both predictions as well as the prediction of crosses that have not been performed.

In the case of similar magnitudes of NSH and BSH, there is no need for dominance adjustment, and only half-sibs can be used. In *M. maximus*, the estimated NSH and BSH for important traits showed a remarkably low and high magnitude, respectively, based on the use of phenotypic data (Simeão-Resende et al., 2004) or genomic data (Lara et al., 2019). In this species, GS based on additive and dominant effects needs to be performed to obtain the highest levels of genetic gain. In this way, it is important to work with tetrasomic inheritance more than disomic inheritance (Lara et al., 2019) and more genetically diverse synthetic populations to elevate the heritability and accuracy of GEBV prediction. The higher dominance effect evidenced in tetraploid *M. maximus* cannot be simply extended to other species unless the effect is previously known, or simply tested by different models of GS. de Bem Oliveira et al. (2019) predicted GEBV in blueberries by comparing diploid (data coded as 0, 1, and 2), tetraploid (data coded as 0, 1, 2, 3, and 4), and continuous (data coded as continuous parameterization assuming values between 0 and 1 and a cumulative additive effect) data models at the individual level. The researchers concluded that the use of continuous data generated estimated genetic gain values that were not significantly different from the best models of all traits. As diploid and tetraploid inferences of data did not affect the predictive ability, we can infer that simplified models can perform adequately.

#### Ridge, Bayes, and Lasso Methods

Bayesian methods are associated with systems of nonlinear equations, and non-linear predictions can be more efficient when the QTL effects are not normally distributed owing to the presence of genes with major effects. The linear predictions associated with RR-BLUP assume that all markers with the same allele frequency contribute equally to genetic variation (lack of genes with major effects). In Bayesian estimation, the shrinkage of effect estimates for the model is controlled by the *a priori* distribution assumed for these effects. Different distributions produce different shrinkages. Methods for penalized and Bayesian estimation may include (BayesB, Fast BayesB, BayesCπ, BayesDπ, Lasso, BLasso, and IBLasso) or lack (RR-BLUP, EN, RR-BLUP-Het, and BayesA) direct covariable selection. Bayesian methods are more efficient when the distribution of QTL effects is leptokurtic (positive kurtosis) because of the presence of genes with large effects. The RR-BLUP method is equally efficient when the QTL effects are normally distributed.

Comparisons among the methods for predicting genomic breeding values have been performed. Meuwissen et al. (2001) concluded that the BayesB method is theoretically best because it is slightly superior to RR-BLUP. However, the author simulated genotypic data with the same *a priori* distribution used for the estimation. This approach yielded greater accuracy for this method, although, such accuracy is unattainable in practice if the actual distribution associated with genetic effects differs from the *a priori* distribution assumed for analysis. In general, there is no method that is best under any circumstances because each method may yield significantly different results depending on the population structure and nature of the trait. However, the results obtained by Guo et al. (2012) indicate that the RR-BLUP method is easier to apply and equal to or better than the others for most applications in plants.

**FIGURE 4:**
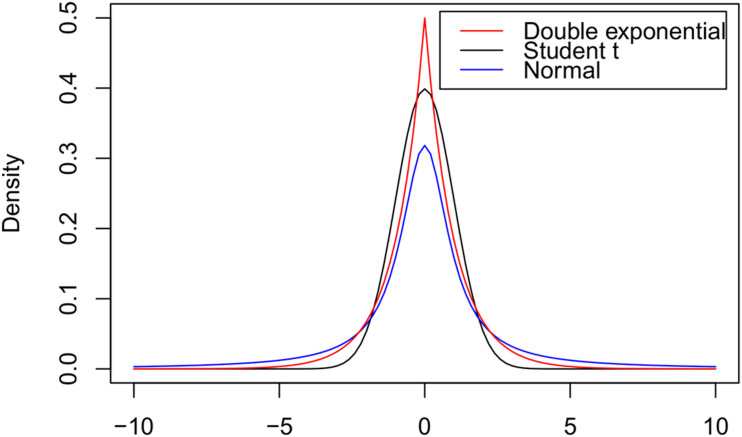
Probability density functions of the double exponential, Student’s t, and normal distributions, all with means equal to zero and variances equal to the unit.

The assumed distributions for the genetic effects of markers in the different GS methods are Gaussian normal with common variance for RR-BLUP, Student’s t-distribution given chi-square priori for variances for Bayesian methods, and Double Laplace exponential for Lasso. Figure 4 illustrates the forms of the normal (RR-BLUP), t (BayesA), and double exponential (Lasso) distributions.

It is observed that, in relation to RR-BLUP, the prior density used in Bayesian Lasso shows a greater density mass at zero point and more robust tails providing greater shrinkage on regression coefficients close to zero and lower shrinkage on regression coefficients away from zero. The prior density used in BayesA also has a higher density mass at the zero point and more robust tails than the normally used RR-BLUP. Bayesian Lasso has greater shrinkage on regression coefficients close to zero than BayesA. However, the distribution tails are similar between the two methods (Figure 4).

The BayesA method implies a large number of markers with small effects or a few markers with moderate to large effects. BLasso implies a large number of markers with effects close to zero or a few markers with moderate to large effects. RR-BLUP implies a large number of markers with small effects.

#### Deep Learning

Machine-learning algorithms (random forest, bagging, support vector machine, and others) have been successful in recognizing complex patterns and making correct decisions based on data. Machine learning is a science of creating and studying algorithms that improve their own behavior in an iterative manner by design (Beysolow, 2017). Recent developments in machine learning enable the implementation of high-dimensional regression using nonlinear methods (Bellot et al., 2018). Another class of models, indeed a subfield of machine learning that became more used to prediction in recent times is deep learning. This theory is devoted to building algorithms that explain and learn a high and low level of abstractions of data that traditional machine learning algorithms often cannot (Beysolow, 2017).

Bellot et al. (2018) present an application of deep learning for the prediction of complex traits comparing the Multilayer Perceptron (MLP) and Convolutional Neural Network (CNN) with commonly used linear regression methods (BayesB and BayesRR). The deep learning under the linear regression, in some cases was very competitive. The MLP and CNN are very heterogeneous classes of predictor that depend on the number of layers, number of neurons per layer, and the activation function. However, the predictive accuracy of Bayesian linear methods is highly dependent on the heritability and this is not a main factor in MLP and CNN.

#### GWAS via the BayesCπ and BayesDπ Methods

Gene discovery or GWAS, which can be accomplished by the BayesCπ and BayesDπ methods (described by Habier et al., 2011), are advantageous because they provide information on the genetic architecture of the quantitative trait and identify the QTL positions by modeling the frequencies of SNPs with nonzero effects. They are advantageous over the regression analysis of single markers because they simultaneously account for all markers. However, care needs to be taken whenever the number of markers is larger than the number of individuals genotyped and phenotyped. Gianola (2013) showed that in such cases, the prior in Bayesian approaches such as BayesC and BayesD, is always influential, which could affect the inference of whether a marker is associated with the trait.

In the BayesC method, a common variance is specified for all loci. The BayesD method maintains the specific variances for each locus. Additionally, π is treated as an unknown with a uniform *a priori* distribution (0,1), thus producing the BayesCπ and BayesDπ methods. The modeling of π is interesting in the association analysis. The majority of the markers are not in LD with the genes; therefore, a set of markers associated with a trait must be identified. In contrast, the BayesB method determines π subjectively. Using the indicator variable δ_*i*_, the BayesCπ and BayesDπ methods model the additive genetic effect of individual j as $a_{j}=\sum_{i=1}^{n}\beta_{i}\,x_{i\,j}\,\delta_{i}$, where δ_*i*_ = (0,1). The distribution of δ=(δ_1_,δ_*n*_) is binomial with a probability of π. This mixed model is more parsimonious than the BayesB method. According to the model hierarchy, a distribution must be postulated for π, and there must be a beta distribution, which when appropriately specified, becomes a uniform distribution (0,1) (Legarra et al., 2011).

The quantities for *x*_*ij*_ are elements of the codominant marker genotype vector and are generally coded as 0, 1 or 2, depending on the number of copies of one of the alleles at the marker locus i, and β_*i*_ is defined as the element of the vector of the regression coefficients, which includes the marker effects on a phenotypic trait *y* by means of the LD with the genes that control the trait (Resende et al., 2013).

#### Sample Size for GS and GWAS

Genomic data are especially useful for GS, which allows selection at the seedling stage to increase genetic gain in the adult stage. With a high density of markers, the expected squared accuracy of GS is given by Daetwyler et al. (2008); Resende et al. (2008); Goddard et al. (2011); Grattapaglia and Resende (2011)$r_{\hat{g}\,g}^{2}=\frac{N\,h^{2}}{\left(N\,h^{2}+n_{Q\,T\,L}\right)}=\frac{N\,h^{2}}{\left(N\,h^{2}+m_{e}\right)}=\frac{N\,h^{2}}{\left(N\,h^{2}+2\,N_{e}\,L\right)}=\frac{N\,h^{2}}{\left(N\,h^{2}+\frac{L}{F}\right)}$, where *N* is the number of genotyped and phenotyped individuals, *L* is the genome size (in Morgans) of the species, *m_e_* is the number of independent chromosomal segments, *Ne* is the effective population size, and *F* is the inbreeding coefficient of the population. For a desired $r_{\hat{g}\,g}^{2}$, *h*^2^, and *n*_*QTL*_, *N* can be determined.

The reliability of GS is given by the expression $r_{g\,g}^{2}=\frac{N\,h^{2}}{N\,h^{2}+N_{Q\,T\,L}}$, where *r*_*gg*_ equals GS accuracy, *N* is the number of individuals in the population, *N*_*QTL*_ is the number of QTLs that control each trait, and *h*^2^ is the individual heritability. The estimate of the number of individuals that must be evaluated to obtain the desired accuracy can be obtained by the following expression, derived from the previous one, $N=\frac{r_{g\,g}^{2}\,N_{Q\,T\,L}}{\left(1-r_{g\,g}^{2}\right)\,h^{2}}$ (Resende et al., 2014).

**FIGURE 5:**
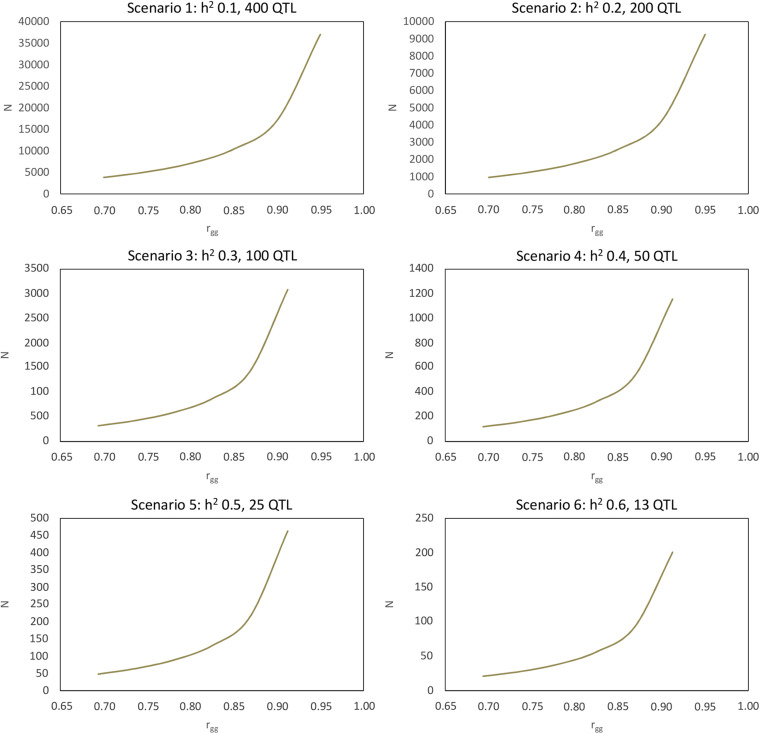
Sample size for genomic selection with desired accuracy ranging from 0.70 to 0.95 in six scenarios in terms of heritability and quantitative trait locus (QTL) number.

Figure 5 shows the curve graphs with N in various scenarios (functions of h^2^, N_*QTL*_, and r_*gg*_). Based on these graphs and the genetic information of the traits, breeders can adequately size their studies on inheritance and maximize genetic gain with the improvement made by selection.

Various kinds of information can be obtained from Figure 5. For example, considering scenario 3, it appears that for a trait with individual heritability equal to 0.30 and that is controlled by 100 QTLs, an accuracy of 90% can be obtained if the sample size is equal to 1,500 genotyped and phenotyped individuals.

From the first equation, the estimate of the number of QTLs that control each trait can be calculated based on the expression $N_{Q\,T\,L}=\frac{\left(1-r_{g\,g}^{2}\right)\,N\,h^{2}}{r_{g\,g}^{2}}$. Once the selective accuracy and heritability are estimated, given the N practiced in a study, the *N*_*QTL*_ can be estimated for several traits.

**FIGURE 6:**
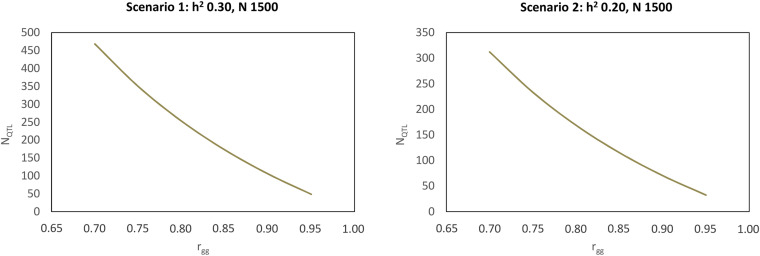
Number of quantitative trait loci (N_*QTL*_) for genomic selection with accuracy ranging from 0.70 to 0.95 in two scenarios in terms of heritability and individual sample size.

A possible exercise is the theoretical determination of N_*QTL*_, given the N and the estimated h^2^ while varying $r_{g\,g}^{2}$. For a case of h^2^ equal to 0.30, and N equal to 1,500, the N_*QTL*_ values can be inferred according to Figure 6. The same figure shows the case of h^2^ equal to 0.20, and N equal to 1,500.

Based on Figure 6, it can be seen that for N = 1,500 genotyped individuals, the QTL numbers vary from 49 to 468 (Scenario 1, h^2^ = 0.30) and from 32 to 312 (Scenario 2, h^2^ = 0.20), when the accuracy varies from 0.95 to 0.70, respectively.

#### Sample Size for Gene Detection

The sample size (N), with power of detection at a significance level of 10^–5^ according to the $h_{m\,i}^{2}$ magnitude of the QTL (considered as random effect), is given by Resende (2015)$N\approx \frac{{\left(Z_{\left(1-\frac{\alpha }{2}\right)}+Z_{\left(1-\beta \right)}\right)}^{2}\,\left(1-h^{2}\right)}{h_{m\,i}^{2}}$, where $Z_{\left(1-\frac{\text{α}}{2}\right)}$ and *Z*_*1*–β_ are the values of the cumulative distribution function of the standard normal distribution, associated with the probabilities of error type I (α) and error type II (β) for bilateral hypothesis tests.

The quantity (1−β) is the probability that the experiment will exhibit a statistically significant difference between the treatment averages. Values of 0.80 and 0.90 are common and appropriate in practice.

**FIGURE 7:**
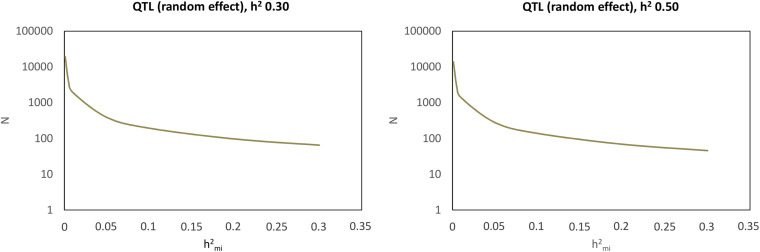
Sample size (N) required to detect genetic effects of markers (assumed to be random effects) with different marker heritability (h^2^_*mi*_) and total heritability (h^2^): N values as a function of h^2^_*mi*_. The plotted N values were obtained via logarithmic transformation to improve visualization.

Table 2 and Figure 7 show that the sample sizes (<1,000) commonly used in plant breeding only detect QTL when the QTL explains 5% or more of the phenotypic variation, a fact that is unlikely under polygenic inheritance (total trait h^2^ <0.50). The power of 0.90 is more appropriate because it leads to an 81% = 0.90^2^ probability that two independent studies will detect the same QTL.

**TABLE 2:** Sample size (*N*) and power for detection of significance level 10^–5^ according to the $h_{m\,i}^{2}$ magnitude of the quantitative trait locus, considered as having a random effect: $N\approx \frac{{\left(Z_{\left(1-\frac{\alpha }{2}\right)}+Z_{\left(1-\beta \right)}\right)}^{2}\,\left(1-h^{2}\right)}{h_{m\,i}^{2}}$.

h^2^ = 0.30					h^2^ = 0.50				
	
Z for β = 0.90	Z for α = 10^–5^	${\left({\text{Z}}_{\left(1-\frac{\alpha }{2}\right)}+{\text{Z}}_{\left(1-\beta \right)}\right)}^{2}$	${\text{h}}_{m\,i}^{2}$	*N*	Z for β = 0.90	Z for α = 10^–5^	${\left({\text{Z}}_{\left(1-\frac{\alpha }{2}\right)}+{\text{Z}}_{\left(1-\beta \right)}\right)}^{2}$	${\text{h}}_{m\,i}^{2}$	*N*
1.28	3.99	27.7729	0.001	19441	1.28	3.99	27.7729	0.001	13886
1.28	3.99	27.7729	0.005	3888	1.28	3.99	27.7729	0.005	2777
1.28	3.99	27.7729	0.01	1944	1.28	3.99	27.7729	0.01	1389
1.28	3.99	27.7729	0.05	389	1.28	3.99	27.7729	0.05	278
1.28	3.99	27.7729	0.1	194	1.28	3.99	27.7729	0.1	139
1.28	3.99	27.7729	0.2	97	1.28	3.99	27.7729	0.2	69
1.28	3.99	27.7729	0.3	65	1.28	3.99	27.7729	0.3	46

## GS Applications in Brachiaria, Guinea Grass and Elephant Grass Breeding

In TFG breeding, combining conventional breeding efforts and GS has not been a simple task. As opposed to advances in animal breeding and crop commodities, they have been slow and challenging in tropical forages. The number of candidate TFG species is high, and decisions about investments need to be made considering the effective benefits of GS, the potential profit that can be achieved by the new cultivars, and the real impact of new forage on livestock production. The three tropical genus/species brachiaria (*Urochloa* spp.), Guinea grass (*M. maximus*) and elephant grass (*C. purpureus*) are very important and extensively used as pastures in tropical America, Asia, and sub-Saharan Africa.

Marker number and density are important factors influencing the efficient use of GS in TFG breeding. One of the reasons for the low accuracy of GS is the exceptionally low number of effective markers, which may result from a non-adequate reference genome. Before the *U. ruziziensis* genome assembly was publicly available, the genomes of *S. viridis* and *P. virgatum* were frequently used as reference genomes for SNP calling and linkage map construction in *Urochloa* species with 1,000 SNPs (Ferreira et al., 2019) and *M. maximus* with 1,322 SNPs (Deo et al., 2020). For *C. purpureus*, 20,144 SilicoDArT and 28,610 SNP markers have been mapped onto the pearl millet (*C. americanus*) reference (Muktar et al., 2019). Compared with other agronomically important *Poaceae* species, such as maize, for which the 50 K Illumina MaizeSNP50 BeadChip (Ganal et al., 2011) and the 600 K Affymetrix Axiom Maize Genotyping Array (Unterseer et al., 2014) are available, the number of markers in TFG needs to be significantly enhanced. Genome calling using reference genomes of other grasses improves the number of SNPs, as shown by Matias et al. (2019a), who reported >26k SNPs in *Urochloa* hybrids. However, the minimum allele depth used was ≤2 reads considering allele dosage and resulted in a low predictive ability (<0.31) in GS for agronomic traits. Similar results were obtained by Lara et al. (2019) in *M. maximus* in which >32k SNPs were classified as unique and used in GS, although, the maximum value of predictive ability using tetraploid dosage of 0.3955 was achieved for the trait organic matter that displayed secondary importance in forage breeding.

Current TFG breeding programs lack the important information that could improve and allow the efficient use of GS. Firstly, a major impact will be obtained by increasing the number of markers per genome size by sequencing and generating reference genomes for the target species or more closely related species. Secondly, we need to improve our knowledge about the inheritance of target traits in tropical forages including the genetic effects of biallelism in (auto) tetraploids. Thirdly, we need to work with training populations connected with validation and breeding populations and testing environments that must be correlated with the environment of the target population (Burgueño et al., 2012; Jarquín et al., 2014; Santantonio et al., 2020). Finally, we need to work with half-sib and full-sib progenies to improve the predictive ability for traits in which the dominance effects are significant, aiming to predict crosses that are not performed in tetraploid *Urochloa* and *M. maximus* hybrid development programs.

The current *M. maximus* and *Urochloa* breeding programs generate thousands of hybrids annually, and those hybrids must pass through several steps of selection until they finally achieve the status for evaluation under animal feeding pressure to prove their value in animal production and be released as new cultivars. However, it is mandatory for hybrids to show apomixis and resistance/tolerance to spittlebugs (mostly in *Urochloa*) and diseases (mostly in *M. maximus* and *C. purpureus*). These traits are a great bottleneck slowing the subsequent evaluation steps in the breeding program since phenotyping of individuals demands significant labor, time (two or more years), and the dedication of trained technicians. As a result, only a small number of individuals can be evaluated annually reducing the rate of genetic gain. Methods such as GWAS and MAS may help to speed up the identification of individuals showing these important traits and increased rates of genetic gain. Recently published data on mapping genomic regions associated with apospory emphasize the routine application of markers for selection in *Urochloa* and *Megathyrsus* (Worthington et al., 2016; Deo et al., 2020).

Finally, TFG breeding programs can be sped up by the application of GS. However, this demands greater investments; collaboration among breeders, molecular biologists, and bioinformaticians; integration of research teams from different institutions and countries; and most importantly, continuity associated with critical course corrections.

## Additional Methods Applied to TFG Breeding

The use of new efficient high-throughput methodologies in addition to GS should be discussed according to their accuracies and potential use in higher numbers of individuals at initial stages of selection. The first obvious application is its use when no genotyping tool is available at a reasonable cost. A second application would be to use phenomics to screen nearly fixed genetic materials which is likely to capture non-additive genetic effects. Nonetheless, phenomics could deliver breeding innovations, and the challenge represented by the breeding target scenario (Reynolds et al., 2020).

Phenomic selection (PS) using high-throughput phenotyping methods less expensive than genotyping by sequencing is an opportunity for tropical forage breeding. Rincent et al. (2018) proposed using near-infrared spectroscopy (NIRS) variables generated as regressors or to estimate kinship in the same statistical models used in GS to perform PS. The results were promising and cost affordable for wheat and poplar when compared to GS. TFGs are a probable candidate for this method because phenotyping using NIRS to obtain bromatological data is routine in research programs and may be studied and amplified to other spectra to be performed as a routine method of PS. Biomass measuring in TFG is a laborious, time-consuming, and biased task, because of the necessity of several annual evaluations (4 to 7) during selection. It also limits the number of individuals in experiments (300 to 2,000). Sensor-based images enabled high-throughput non-invasive phenotyping throughout the growing cycles of forage grasses, and the models established a high correlation between images and the biomass yield in *M. maximus* (Castro et al., 2020) as well as for crude protein percentage and chlorophyll concentration in *Urochloa* (Jiménez et al., 2020). Deep learning-based neural network studies demonstrated that accuracies must be increased by pre-trained models and data augmentation (Castro et al., 2020). Nevertheless, deep learning progress is accelerating and will be able to perform better predictions than ever (Montesinos-López et al., 2021). Although it has been the subject of debate in the past, extra investment in phenotyping technologies is becoming more accepted to capitalize on recent developments in crop genomics and prediction models. In this context the different strategies for phenotyping can be built from phenomic selection (Rincent et al., 2018), high-throughput phenotyping, and detailed characterization or ‘precision’ phenotyping (Reynolds et al., 2020).

## The Future of GS in Forage Breeding

The availability of genome-wide, high-throughput, and cost-effective flexible markers, across the genome, suitable for large populations with or without a reference genome sequence, is the most important factor for the effective and efficient implementation of GS. Recent advances in long read quality and sequence throughput, in addition to other technologies such as Hi-C or optical maps, make it possible for virtually any research group with reasonable funding to obtain reference-grade genome assemblies for their crop of choice. While not necessarily easy, the generation of high-quality genome assemblies should be considered as a starting point for any orphan species that would benefit from the use of genomic tools for crop improvement. These assemblies could be extremely useful for resequencing and variant discovery, which can lead to genotyping platforms for association studies and GS. When coupled with well-designed and thoroughly phenotyped training populations, these genomic resources could serve as the basis for implementing GS steps in the breeding of TFGs.

As discussed by Lin et al. (2014) and Bhat et al. (2016), the cost of identifying and genotyping a large number of SNPs is still a barrier for TFGs, although, second-generation sequencing technology has provided new SNP genotyping platforms, particularly GBS. In addition, phenotyping large representative reference populations is expensive. Reduction of phenotype assessment costs per individual and new phenomic approaches are essential to take advantage of the true benefits of GS. Marker technologies must be combined with high-throughput phenotyping to achieve significant genetic gains for complex traits.

Furthermore, the considerations stated by Simeão-Resende et al. (2014) are still valid. GS will allow an increase in the early-generation of number of individuals evaluated considering the large number of targeted traits. However, when we deal with GS in the improvement of tropical forages, we realize that there is still a long way to go. Theoretically, by models and methods already developed and successfully applied in commodity species, the procedures could be easily incorporated into the routine of breeding programs. Nevertheless, in orphan species, all knowledge needs to be built on solid molecular bases. In principle, the evaluation of a large number of individuals for selection purposes increases the probability of the best allelic combinations for traits of economic importance without narrowing the genetic basis for selection. This should be considered in the improvement of polyploid and apomictic *Urochloa* spp. and *M. maximus*. Performing inter- and intraspecific crosses with sexual plants in these genera increases the variability available for selection and allows the generation of genetic combinations not found in apomictic accessions. The spectrum of possibilities for GS expands considerably for these species; however, the identification of markers is narrowed to large-scale phenotyping and genotyping. The conformation of the discovery population should be carefully considered in terms of the number of hybrid families to be evaluated, the number of individuals per half-sib and full-sib families, and the distribution of markers on the chromosomes of the paternal and maternal genomes. The mother plants to be used in crosses should be exclusively sexual, so that they do not generate, in addition to hybrids, their own clones (by apomixis) in the progeny, which would cause an incorrect bias in the determination of GEBV and the identification of markers and their effects.

Finally, designing forage breeding programs, mainly for polyploid and apomictic grasses, and proposing breeding schemes that make optimum use of GS is a significant task for plant breeders. Although, this is a challenge, it is also a great opportunity to accelerate genetic gain in TFG breeding.

## Author Contributions

All authors listed have made a substantial, direct and intellectual contribution to the work, and approved it for publication.

## Conflict of Interest

The authors declare that this review was written in the absence of any commercial or financial relationships that could be construed as a potential conflict of interest.

## Abbreviations

BLUP: best linear unbiased prediction
CIAT: International Center for Tropical Agriculture
EMBRAPA: Empresa Brasileira de Pesquisa Agropecuária
GBS: genotyping-by-sequencing
GEBV: genomic breeding values
GETV: total (genotypic) genomic values
GLS: generalized least squares
GS: genomic selection
GV: genomic value
GWAS: genome-wide association studies
IBERS: Institute of Biology Environmental and Rural Sciences at Aberystwyth University
ICARDA: International Center for Agricultural Research in the Dry Areas
ILRI: International Livestock Research Institute
LD: linkage disequilibrium
NIRS: near-infrared spectroscopy
MAF: minor allele frequency
MAS: marker-assisted selection
QTL: quantitative trait loci
QTN: quantitative trait nucleotide
REML: restricted maximum likelihood
SARDI: South Australian Research and Development Institute
SNP: single-nucleotide polymorphism
TFG: tropical forage grass
USDA: United States Department of Agriculture.
